# Sustainable gelatine–alginate biopolymer binder enhancing pigment printing and functional performance of textiles

**DOI:** 10.1038/s41598-025-26530-y

**Published:** 2025-11-24

**Authors:** Nermin A. Ibrahim, Heba M. El-Hennawi, Safia A. Mahmoud, Ahmed G. Hassabo

**Affiliations:** 1 Textile Printing, Dyeing and Finishing Department, Faculty of Applied Arts, Beni-Suef University, Beni-Suef, Egypt; 2 Dyeing, Printing, and Intermediate Auxiliaries Department, National Research Centre, Textile Research and Technology Institute, 33 El-Behouth St. (Former El-Tahrir Str.), Dokki, P.O. 12622, Giza, Egypt; 3 Pre-Treatment, and Finishing of Cellulose-Based Textiles Department, National Research Centre, Textile Research and Technology Institute, 33 El-Behouth St. (Former El-Tahrir Str.), Dokki, P.O 12622, Giza, Egypt

**Keywords:** Gelatine, Eco-friendly Binder, Pigment, Textile Printing, Biotechnology, Materials science

## Abstract

This study investigates gelatine and sodium alginate as novel thickening agents for pigment textile printing using natural eco-friendly binders. As a thickener and binder, gelatine was tested in different ratios to sodium alginate. The study found that shear rate significantly affected gelatine gel-based thickener shear stress and viscosity. The study examined gelatine-based thickening agents’ elasticity and performance when thinned. The study found that adding sodium alginate increases apparent viscosity compared to gelatine gel alone. A 50:50 gelatine-alginate thickener ratio produces optimal printing paste viscosity. The samples with gelatine and sodium alginate had the highest K/S, while those without gelatine had the lowest. The study tested printed fabrics for light, washing, sweat, and rubbing fastness. All printed fabrics with pigment and gelatine/alginate (1:1) thickener had deeper color depth. After 15 days at ambient temperature, gelatine-based thickening printed fabrics were tested for antibacterial activity against Escherichia coli, Staphylococcus aureus, and Candida albicans.

## Introduction

One of the most essential ways to color fabrics in the industry is through textile printing. This approach can make complicated designs and functional finishes on materials. Typical pigment printing systems use synthetic binders and thickeners like polyacrylates or polyvinyl alcohol. These work well for color yield and durability, but they also have big environmental problems, like being hard to break down, releasing volatile organic compounds, and polluting wastewater^1–7^. As the world becomes more focused on sustainability and eco-efficiency, more and more people are interested in replacing traditional auxiliaries with bio-based, renewable ones^8–12^.

Natural polymers including starch, guar gum, sodium alginate, and chitosan are already being used in printing and finishing because they are safe, biodegradable, and easy to find^13–17^. Alginate, derived from brown seaweed, is extensively utilized as a thickening in textile printing due to its superior film-forming properties and solubility^18^. Conversely, gelatin, which comes from collagen, has been studied for its ability to gel and produce films, but its use in pigment printing is not as well known^5,14,16,19^.

Recent studies have shown that bio-based thickeners can give textiles multiple functions. Chitosan and its derivatives have dual functions as printing thickeners and as enhancers of antibacterial activity. Natural polymers containing aromatic or heterocyclic groups can likewise enhance UV protection in textiles. However, much past research has concentrated on single natural thickeners, with little investigation into synergistic systems that integrate protein- and polysaccharide-based polymers to evaluate rheology, adhesion, and functionality.

Gelatin is a high molecular weight, biodegradable, and non-toxic polypeptide^5,14,16,19–21^. Gelatin is a water-soluble protein derived from the partial hydrolysis of collagen. The principal fibrous protein present in bones, cartilage, and skin. The source, age of the animal, and type of collagen are intrinsic factors determining the properties of gelatins^22,23^.

Acidic or alkaline baths and thermal pre-treatments are employed to extract proteins from skin and bone during the production of gelatine. A heating process is subsequently employed to isolate the proteins from the remaining raw material^24^. The extract is subsequently dried, sterilized, and deionized; however, other stages may be incorporated based on the production process. The resultant dried substance is referred to as gelatin. Gelatine is generally formed in two forms: type A (acid hydrolysis) and type B (alkaline hydrolysis), contingent upon the employed technology^22,25^.

The quantity and quality of its applications have risen over time as its production has grown more industrialized^24^. It is being utilized extensively across various industries, including textiles, food, pharmaceuticals, and photography^25^. Gelatine can improve pretreatment processes such as scouring and bleaching by reducing temperature, minimizing alkali usage, and enhancing the efficacy of the bleaching method. Furthermore, gelatine may be included to enhance the dyeability of the material. It is relevant to the processing of textiles to produce multifunctional textiles^26–29^. This study investigates the feasibility of utilizing gelatine as a thickening agent and a natural, eco-friendly binder for pigment-based textile printing.

The most direct method for coloring textiles is pigment printing, which utilizes insoluble organic or inorganic compounds that are adhered with binding agents but lack affinity for textile substrates^30–33^. Nonetheless, conventional synthetic binders sourced from petroleum are carcinogenic, often emit volatile organic compounds (VOCs) during production, and are detrimental to human skin. Numerous studies have focused on natural binders such as chitosan and modified wheat gluten, addressing the increasing demand for eco-friendly binders with excellent color fastness in recent years^34,35^.

The study presents an innovative eco-friendly thickening and binding technique that integrates gelatin, a protein-based natural polymer, with alginate, a polysaccharide, for pigment printing on textiles. This system is biodegradable, renewable, and non-toxic, which is in line with developments in sustainable textile processing. This is different from traditional synthetic binders and thickeners like acrylics and PVA. The study uniquely assesses various performance attributes concurrently: rheological behavior, printability, color strength (K/S), fastness qualities, UV protection, and antibacterial efficacy. It is also one of the first studies to look into how varied ratios of gelatine to alginate affect the printing performance and functional qualities of fabrics that have been printed on.

The primary objective of the project is to create and assess an environmentally sustainable gelatin-alginate binder system for pigment printing, intended to supplant traditional synthetic binders. The study’s specific objectives are to: (a) study how varied amounts of gelatine and alginate affect the flow of the substance, (b) check how these binders affect the color strength (K/S values) and fastness of printed cotton, polyester, and mixes, (c) Look at the extra functional benefits of printed fabrics, such how they protect against UV rays and kill germs, and (d) find the best formulation that strikes a balance between print quality, fabric durability, and environmental friendliness.

In this context, the current work examines the application of a gelatine–alginate hybrid binder system for environmentally sustainable pigment printing on cotton, polyester, and blended textiles. By changing the ratio of gelatine to alginate, the study methodically looks at how it affects rheological qualities, print quality, color strength (K/S values), fastness performance, and other features like UV protection and antibacterial activities. This method gives us fresh information on how to combine biopolymers to improve both printing performance and sustainability. It could be a good replacement for traditional synthetic binders.

## Experimental

### Materials

Cotton, Polyester and Cotton/Polyester 50/50 in which cotton fabric was 153 g/m^2^ and polyester fabric was 215 g/m^2^, Cotton/Polyester 50/50 fabric s 198 g/m^2^ fabrics provided by Textile Industries Egyptian Co. Ointex, Egypt. Gelatine and sodium alginate were purchased from Loba Co. India.

**Fig. 1:**
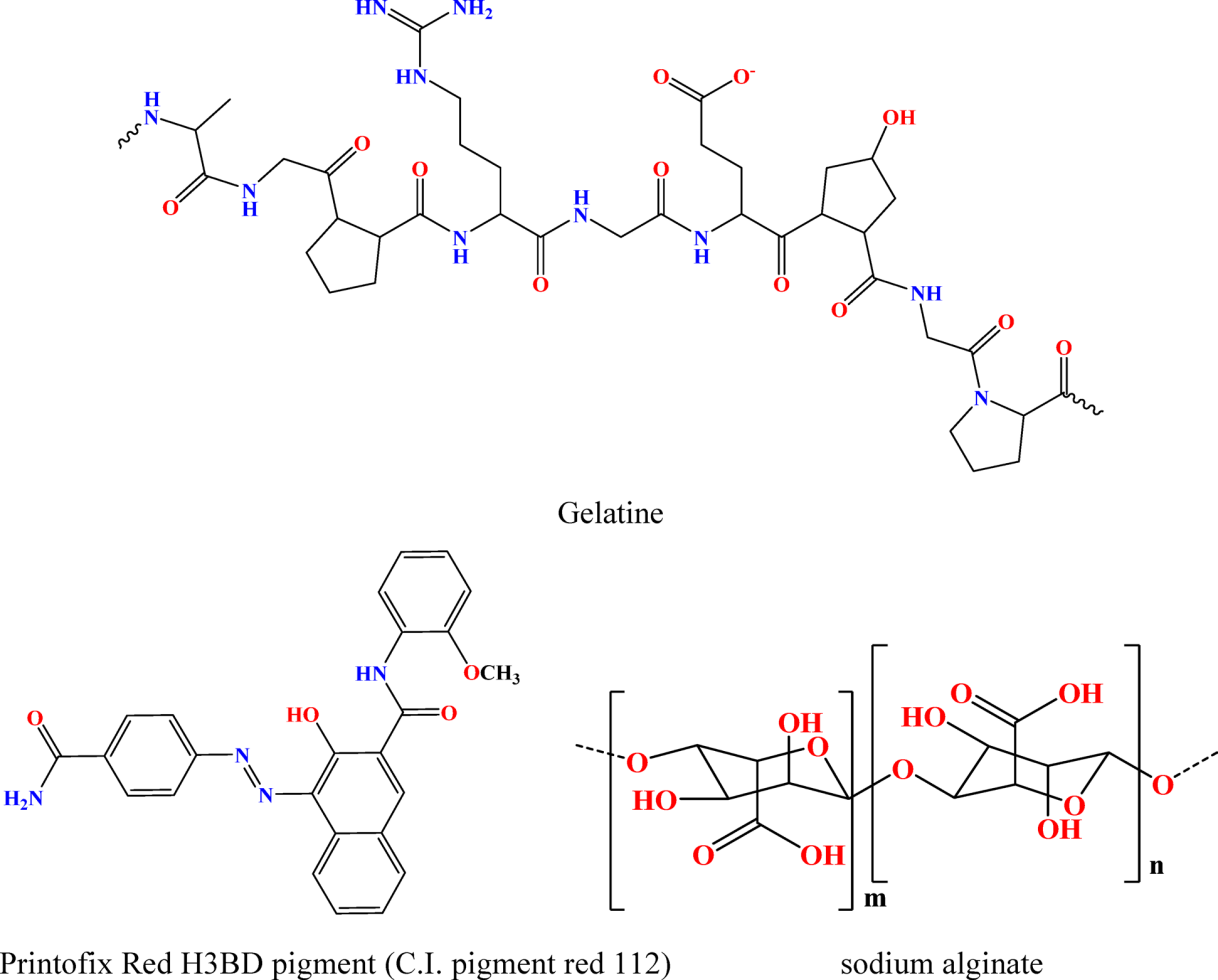
Chemical structure of Gelatine and pigment.

Printofix Red H3BD pigment, synthetic binder Printofix MTB (acrylate-based copolymer, anionic) supplied by Clarient, were used in the conventional pigment printing. the chemical structure is shown in Fig. 1.

### Procedures

#### Screen printing

Three distinct fabric types (cotton, polyester, and a 50/50 cotton/polyester blend) were printed using flat-screen technology with variable pigment concentrations (1%, 2%, and 3%) and different ratios of gelatin to sodium alginate, specifically: gelatin, 1:4, 2:1, 1:1, 1:2, 4:1, and sodium alginate. The standard printing paste utilized in this experiment is presented in Table 1; this paste was also employed to print the blank samples. Following printing, textiles were dried at 100 °C for 5 min and thermofixed at 160 °C for 4 min. Following fixation, washing was conducted in four stages. Printed fabrics were initially rinsed in cold water for approximately 15 min, followed by a second rinse in warm water at 60 °C for about 15 min, and a third rinse in warm water at 60 °C with a non-ionic detergent for an additional 15 min. Finally, they were thoroughly rinsed in cold water and dried at 85 °C for 5 min^36,37^.

**Table 1:** Formulation of pigment printing paste.

Components of printing paste	Weight/gm
pigment	30–50 gm
Urea	25 gm
Thickener	500 gm
Binder	50 gm
Sodium dihydrogen phosphate dehydrates	5 gm
Distilled water	Y gm up to 1000 gm
Total	1000 gm

### Analysis and measurements

#### FTIR-analysis

FTIR spectra were created for selected samples using a Spectrum 65 FTIR spectrometer (PerkinElmer Co., Ltd., MA, USA).

#### Nitrogen content

The ASTM E258-07 (Reapproved 2015) method was used to determine the nitrogen content^38^.

$${\text{Nitrogen}}\;{\text{content}} = \frac{N \% }{{14}} \times 100\;\left( {{{{\text{mmole}}} \mathord{\left/ {\vphantom {{{\text{mmole}}} {{1}00\;{\text{g}}\;{\text{sample}}}}} \right. \kern-0pt} {{1}00\;{\text{g}}\;{\text{sample}}}}} \right)$$

#### Rheological behavior

The rheological properties of the thickening agent were examined at 25 ± 0.1 °C using a coaxial rotary viscometer (HAAK V20), Germany. In Newton’s law, the apparent viscosity (η; Pascal.second) is defined as the ratio of shear stress (τ; Pascal) to shear rate (γ; s⁻^1^) as expressed in the following equation^39,40^:

$${\varvec{\eta}} = \user2{ }\frac{{\varvec{\tau}}}{{\varvec{\gamma}}}$$

Viscosity can be defined as molecular attraction, namely the internal friction inside the fluid that produces resistance to flow. Viscosity is an essential rheological parameter in textile printing that defines the flow characteristics of thickeners. Textile paste printing is typically categorized as a thixotropic fluid. Thixotropic refers to a substance that seems paste-like when at rest but becomes fluid when subjected to stress^41^.

The viscosity of these pastes decreased with time under a constant shear stress, as shown by the Shear Thinning Index (STI)^42^. The viscosity of the thickening decreases with elevated shear stress, yielding a more flowing pasta. However, without external stress, the paste will maintain its viscosity.

During the printing process, a reduction in shear allows the paste to enter the stencil apertures, while the viscosity of the printing paste increases with the alleviation of shear tension, so enhancing the compound’s capacity to attain its geometrically printed form. An inadequate quantity of a highly viscous paste leads to the enlargement of the joints. Decreased viscosity leads to structural collapse and blockage. Therefore, it is essential to assess the flow properties of thickeners to attain optimal printing performance^10,43–47^.

Viscosity computational models, such as the power-law, can be employed to assess the precision of the processing range and the data available for experimental investigation^48,49^. The viscosities of several thickeners are analyzed at different shear rates.

#### Power law model

The power law model is widely recognized as the Ostwald de Waele power-law equation.

$$\tau = K \gamma^{n}$$

In this context, τ represents shear stress, γ denotes shear rate, and K shows the consistency coefficient that defines the average viscosity distribution over the current flow curve, reflecting the viscosity or stress at a specific shear rate. The variable n represents the power-law index. In a shear-thinning fluid, the n value ranges between 0 and 1 1 (0 < n < 1); thus, the closer a sample is to zero, the more pronounced the shear thinning effect. Viscosity can be defined as follows^48,50,51^:

$${\varvec{\eta}} = \user2{K\gamma }^{n - 1}$$

Graphs of shear stress against shear rate for particular fluids display linearity when presented in logarithmic style. The Power-law model clarifies phenomena related to the thinning and thickening of shear in fluids. Thus, the following equation may be derived by applying the natural logarithm to both sides of the above equation:

$${\text{Log}}\;({\varvec{\eta}}) = \left( {{\text{n}} - {1}} \right)\;{\text{Log}}({\varvec{\gamma}}) \, + {\text{ Log}}({\varvec{K}})$$

This relationship is linear in the logarithm of the plot compared to the logarithm. Nevertheless, it is advantageous for evaluating and scrutinizing trends in experimental results. This model is beneficial since it can handle data within a shear rate range of 10 to 10,000 s^−1^^48^. This model neglects the ramifications of constant viscosity at both low and high shear rates^44,45^.

#### Color assessments

The color intensity of printed fabrics was evaluated using the Hunter Lab Ultra-Scan Pro at the National Research Center in Egypt. The standard format will be designated as K/S. The K/S values were determined using the Kubelka–Munk equation^52–58^.

$${{\text{K}} \mathord{\left/ {\vphantom {{\text{K}} {\text{S}}}} \right. \kern-0pt} {\text{S}}} = \frac{{\left( {1 - {\varvec{R}}} \right)^{2} }}{{2{\varvec{R}}}} - \frac{{\left( {1 - {\varvec{R}}_{{\varvec{o}}} } \right)^{2} }}{{2{\varvec{R}}_{{\varvec{o}}} }}$$

K signifies the absorption coefficient; S represents the dispersion coefficient; R defines the reflectance of the fabric at its maximum wavelength. Three replicates were conducted for each measurement to compute the standard deviation (STD).

CIE Lab values assessed under D65 lighting, 10° standard observer, d/8 geometry, including SCI, observer: CIE 1931 was 2° and background: Neutral gray, approximately 2000 lx.

#### Assessment of color fixation

To evaluate color fixing, dyed materials were washed at 50 °C for 30 min, after which the color strength values of the fabric were assessed both before and after washing. The color fixation percentage (%F) was calculated using the equation shown below^59,60^:

$$\% {\text{F}} = {{\left( {{{\text{K}} \mathord{\left/ {\vphantom {{\text{K}} {\text{S}}}} \right. \kern-0pt} {\text{S}}}} \right)_{{\text{a}}} } \mathord{\left/ {\vphantom {{\left( {{{\text{K}} \mathord{\left/ {\vphantom {{\text{K}} {\text{S}}}} \right. \kern-0pt} {\text{S}}}} \right)_{{\text{a}}} } {\left( {{{\text{K}} \mathord{\left/ {\vphantom {{\text{K}} {\text{S}}}} \right. \kern-0pt} {\text{S}}}} \right)}}} \right. \kern-0pt} {\left( {{{\text{K}} \mathord{\left/ {\vphantom {{\text{K}} {\text{S}}}} \right. \kern-0pt} {\text{S}}}} \right)}}_{{\text{b}}} \times {1}00$$

(K/S)a signifies the color strength of the dyed fabric after washing, while (K/S)b indicates the color strength of the dyed fabric before washing.

#### Characteristics of colorfastness

The colorfastness to washing was evaluated using a Laudner-Ometer, following AATCC test method 61–2013^61^. The color resistance to abrasion (both dry and wet) was evaluated using the Crock Meter, following the AATCC test method 8–2016^62^. The colorfastness to perspiration (acidic and alkaline) was evaluated in accordance with the AATCC test method 15–2013^63^. The evaluation of printed textiles has been standardized using the Gray Scale reference for color variance. The lightfastness was evaluated following AATCC test method 15–2013^64^. The evaluation of the printed products was performed using the blue Scale reference for color modification.

#### Assessments of ultraviolet protection factor (UPF)

The ultraviolet protection factor (UPF) for both untreated and treated cotton fabric samples was assessed according to the Australian/New Zealand standard (AS/NZS 4366–1996). The UV-protecting factor (UPF) of the treated fabrics was analyzed using a UV spectrophotometer using absorption spectroscopy^65^. The control reference was measured as air. The efficacy of UV (UPF) treated cloth was assessed by quantifying the absorption, transmission, and reflection of UV radiation. The UPF value was calculated using the subsequent equation derived from the transmission spectra of the fabric samples in the range of 280–400 nm^66–70^.

$${\text{UPF}} = \frac{{\mathop \smallint \nolimits_{\lambda 2}^{\lambda 1} {\text{E}}\left( \lambda \right) \times {\text{S}}\left( \lambda \right) \times \Delta \left( \lambda \right)}}{{\mathop \smallint \nolimits_{\lambda 1}^{\lambda 2} {\text{E}}\left( \lambda \right) \times {\text{S}}\left( \lambda \right) \times {\text{T}}\left( \lambda \right) \times \Delta \left( \lambda \right)}}$$

The control relation was determined using air, where λ1 and λ2 corresponded to 280 and 400 nm, respectively. E(λ) represents the relative erythemal spectral effectiveness, S(λ) denotes the solar spectral irradiance in W.m^−2^.nm^−1^ (λ) sourced from the National Oceanic and Atmospheric Administration (NOAA) database, T(λ) indicates the spectral transmission of the sample obtained from UV spectrophotometric experiments, and ∆(λ) signifies the difference between measurable wavelengths. Three replicates were conducted for each measurement to compute the standard deviation (STD).

#### Mechanical characteristics of the treated textile

Tensile strength and elongation will be assessed at 25 °C and 65% relative humidity, following ASTM test method D1682-59T, utilizing the FMCW 500 tensile strength equipment (Veb Thuringer Industrie Werk Rauenstein 11/2612 Germany)^71^. The crease recovery angle (CRA) was evaluated following the AATCC test method 66–2014^72^. The SE 1700 surface roughness instrument was employed to assess the roughness of the printed fabrics using ASTM test method D 7127–13. Seventy The rigidity of the printed textiles was evaluated following ASTM test D 1388-14e1 using a cantilever instrument^73,74^. Three replicates were conducted for each measurement to compute the standard deviation (STD).

#### Antibacterial effectiveness

The antibacterial efficacy was quantitatively evaluated against Staphylococcus aureus (ATCC 29213), a gram-positive bacterium, Escherichia coli (ATCC 25922), a gram-negative bacterium, and Candida albicans (ATCC 10231), a fungus, employing the AATCC 100–2004 (bacterial reduction method), a standardized methodological framework for antimicrobial paste assessments^75,76^.

This test is utilized to cultivate a uniform bacterium in liquid medium. The culture is immersed in a sterile nutrition solution. The produced thickening agent is incubated for 24 h at 37 °C with microorganisms in airtight containers. After incubation, agitate for one minute and thereafter evaluate bacterial counts. The initial concentration of bacteria was denoted as a percentage reduction (% R) in microbial volume^77^.

$${\text{Percentagereduction}}\;{\text{of}}\;{\text{bacteria}}\;\left( {{\text{R }}\% } \right) = {{\left( {{\text{B}} - {\text{A}}} \right)} \mathord{\left/ {\vphantom {{\left( {{\text{B}} - {\text{A}}} \right)} {\text{B}}}} \right. \kern-0pt} {\text{B}}} \times {1}000$$

A denotes the number of bacteria obtained from the inoculated test sample in the jar after the designated contact length, whereas B signifies the quantity recovered from the inoculated measured specimen in the jar immediately post-inoculation (at “0” contact time). Three replicates were conducted for each measurement to compute the standard deviation (STD).

#### Handle and sharpness

The texture and sharpness of the printed fabric were evaluated by tactile and visual examination. Three experts evaluated the fabric, and the average of their assessments was recorded. The assessment categorized the fabric handle as soft (S) or harsh (H) and the delineated contour of the printed area as sharp (Sh) or not sharp (NS)^78^.

## Results and discussion

### Characterization of the bio-binder based on gelatine

The main aim of this study was to employ natural eco-friendly binders instead of synthetic binders for pigment textile printing. This study investigated multiple ratio percentages of gelatin to sodium alginate: 100% gelatin, 1:4, 2:1, 1:1, 1:2, 4:1, and 100% alginate. Gelatin was utilized as a thickening and binding agent in this endeavor. The effect of shear rate on shear stress and viscosity of the gelatin gel-based thickener was investigated using alginate (Alg, 7%) at various weight ratios.

**Fig. 2:**
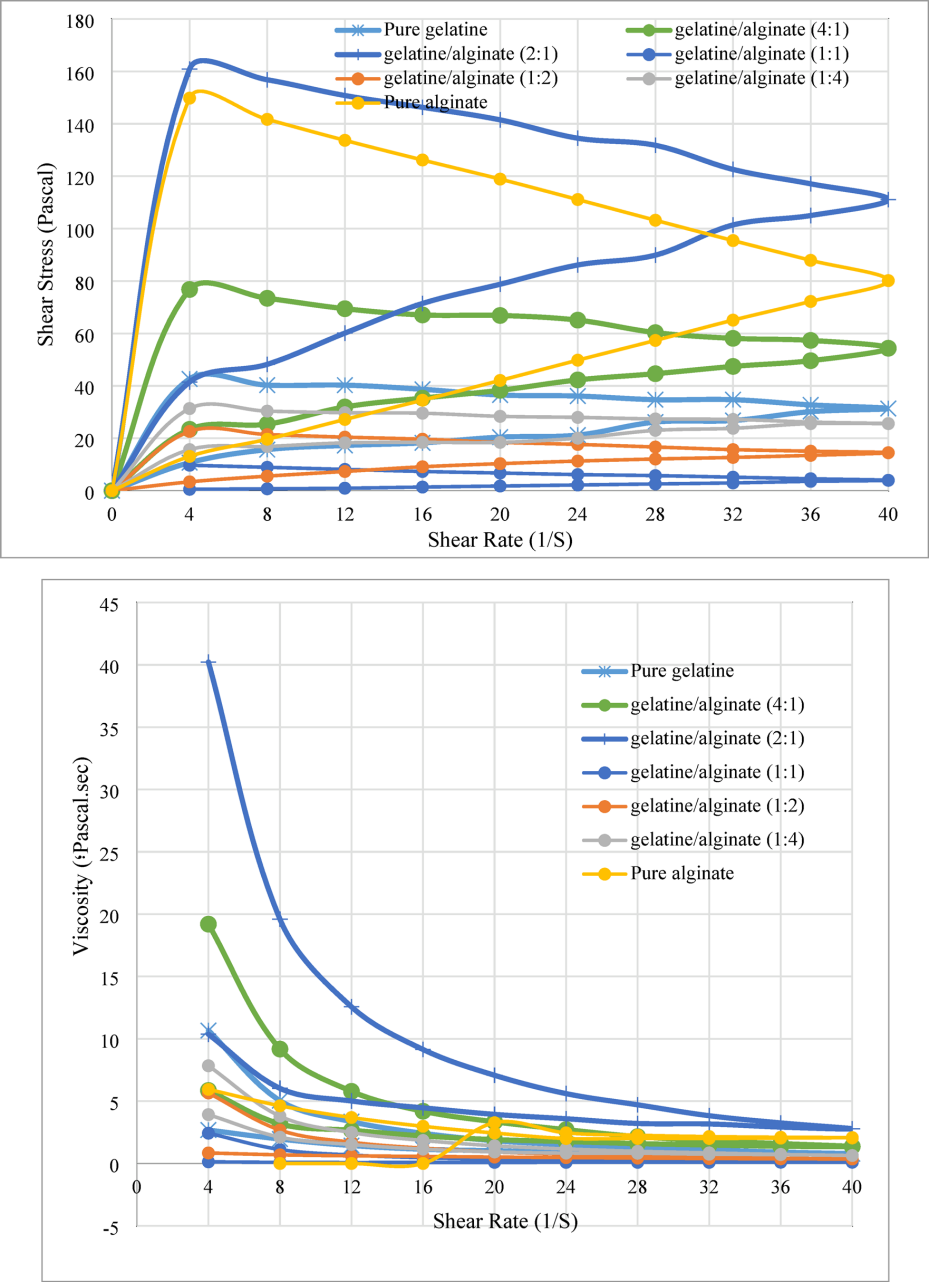
Impact of shear rate on shear stress and apparent viscosity of gelatin thickening agent with alginate at 25 ± 1 °C.

Figure 2 depicts the influence of shear rate on the shear stress of thickening agents, demonstrating that the flow behavior of the formulated thickeners is characterized by the establishment of a hysteresis loop that begins and ends at the beginning point.

These thickening agents are likely to demonstrate non-Newtonian, pseudoplastic shear-thinning behavior, indicating considerable thixotropy^58,78^. The degree of thixotropy (the region between the upper and lower curves)^79,80^ signifies the consistency of the thickening. The thixotropic properties of these thickeners relate to the time necessary for the reconstruction of their internal structures, which have been altered by shear stress.

Thus, it may be concluded that reduced thixotropy is associated with a more elastic thickener, signifying an enhanced capacity of the internal structure to recover after the removal of shear. Thus, the thickening demonstrating significant thixotropy (the region between the upper and lower curves) is recognized as the most pseudoplastic^9^. Table 2 depicts the area between the higher and lower curves for different thickening agents obtained from gelatin, according to the correlations between shear rate and shear stress or viscosity. The gelatine: alginate (1:1) thickener demonstrates minimal area values between the two curves, hence confirming improved elasticity and efficient thickening performance. The negative value of the area between the curves signifies that the return curve (from higher shear rate) exceeds the initial curve (from lower shear rate to higher shear rate).

**Table 2:** Area Region between upper and lower curves for several thickening agents based on gelatine, derived from shear rate vs shear stress graphs.

Thickener	Type of curve	Area under curve (AUC)		Area between curves (Difference)
		Upper curve	Lower curve	
Pure gelatine	Shear stress	809.62	1409.70	− 600.08
	Viscosity	48.51	113.73	− 65.22
Gelatine/alginate (4:1)	Shear stress	1462.14	2486.82	− 1024.68
	Viscosity	202.63	202.63	− 111.78
Gelatine/alginate (1:2)	Shear stress	2952.00	5271.40	− 2319.40
	Viscosity	176.33	− 429.58	− 253.25
Gelatine/alginate (1:1)	Shear stress	79.55	257.97	− 178.43
	Viscosity	3.37	23.28	− 19.92
Gelatine/alginate (1:2)	Shear stress	370.26	699.70	− 329.44
	Viscosity	20.37	58.44	− 38.07
Gelatine/alginate (1:4)	Shear stress	− 771.92	1083.88	− 311.96
	Viscosity	52.23	85.69	− 33.45
Pure alginate	Shear stress	1684.42	4432.18	− 2747.76
	Viscosity	85.70	379.51	− 293.81

The thickener’s maximum resistance to deformation is represented by the ascending path of the loop. This outlines the profile of the maximum apparent viscosity of a specific thickener.

The effect of shear rate on apparent viscosity was also analyzed to understand the thinning behavior of these thickeners. The viscosities of each gelatin gel-based thickener, represented in the upper curves, are quantified in relation to the shear rate, as demonstrated in Fig. 2. Figure 2 illustrates that the apparent viscosity decreases within the observed range as the shear rate escalates. This can be convincingly explained by its pseudo-plasticity, which enables the correct alignment of particles during rotation and reduces their flow resistance^81^.

The apparent viscosity is affected by the gelatin gel, the alginate, and the ratio of the two components.

The increase in viscosity is associated with the higher percentage of the alginate component, indicating that the additive’s function in the gelatin gel enhances viscosity as its proportion rises. The network created by each ingredient improves the surface distribution of gelatin particles, resulting in increased flow resistance and viscosity.

Moreover, the molecular configuration of alginate restricts the mobility of the internal phase due to heightened entanglement and the ratio of gelatin gel, which can be considered a dragging effect.

In conclusion, Fig. 2 demonstrates that increasing the sodium alginate concentration leads to an elevated apparent viscosity compared to gelatin gel alone. A thickener ratio of 50:50 (gelatin: alginate) produces excellent viscosity for printing paste, exceeding the effectiveness of any further increases in the additive proportion within the thickener ratio.

#### Thickener and the power law

The power law clarifies the correlation between shear stress and viscosity about shear stress. This is an empirical relationship that delineates the flow characteristics of fundamental non-Newtonian fluid modes as specified by the subsequent equations^58,78^:

$$\tau = {\mathbf{K}} \times \gamma^{{\mathbf{n}}} \;{\mathbf{and}}\;{\mathbf{h}} = {\mathbf{K}} \times \gamma^{{{\mathbf{n}} - {\mathbf{1}}}}$$

In this context, τ symbolizes shear stress, η represents apparent viscosity, S indicates shear rate, K is defined as non-Newtonian consistency, and n is acknowledged as the Flow Behavior Index (FBI). The power law is characterized by the increasing curves of shear rate in relation to shear stress.

Error! Reference source not found. displays a logarithmic graph of shear stress versus logarithmic shear rate for the top curves of the thickener, revealing linear relationships with varying positive slopes. The slope values, representing the FBI values for the tested thickener in Error! Reference source not found., are all below 1, signifying that the thickeners demonstrate pseudoplastic behavior, as previously established^81,82^. A diminished FBI value improves the pseudo-plasticity of thickening behavior. Error! Reference source not found. displays a logarithmic plot of viscosity versus logarithmic shear rate for the thicker upper curves, which demonstrate straight lines with differing negative slopes. The slope is equivalent to the n–1 slope. The Shearing Thinning Index (STI) for any thickener is defined as the absolute value of the slope, denoted as “1–n.” For each thickening, the quantities of both the FBI and the STI must be exactly 1, with no variations, as demonstrated in Error! Reference source not found.. A diminished STI is associated with improved and more resilient flow characteristics^81,82^.

The likely intermolecular interactions between gelatin and alginate are crucial for improving rheological and printing performance. The electrostatic attraction between the positively and negatively charged amino acid groups in gelatin and the negatively charged carboxyl groups in alginate forms ionic bonds that stabilize the network. The predominance of –NH and –OH groups in gelatin, together with –OH and –COOH groups in alginate, facilitates enhanced stability through hydrogen bonding. The electrostatic interaction between gelatin and alginate, owing to their opposing charges, results in the creation of a dense polymer-rich phase, hence enhancing structural integrity. The entanglement of these long-chain polymer chains enhances viscosity and mechanical strength. Hydrophobic interactions among the hydrophobic amino acid residues of gelatin enhance the overall stability of the gelatin-alginate network. The multiple weak van der Waals interactions between the alginate and gelatin molecules jointly strengthen the cohesiveness of the blend. These interactions enhance rheological properties by augmenting viscosity, optimizing shear-thinning behavior, and facilitating structural recovery under shear, so enhancing printing performance.

### Characterization of the printed fabrics

The main aim of this study was to employ natural, environmentally friendly binders instead of synthetic binders for pigment textile printing. The study sought to evaluate and determine the ideal printing material for producing darker prints with enhanced fastness characteristics. This work utilized gelatine and alginate as innovative thickeners for pigment printing on diverse textile substrates. Three unique fabrics—cotton, polyester, and a cotton/polyester hybrid (50/50)—were printed utilizing three various pigment concentrations (1%, 2%, and 3%) with changing ratios of gelatine to sodium alginate, namely (100% gelatine, 1:4, 2:1, 1:1, 1:2, 4:1, and 100% alginate). Gelatin functioned as both a thickening agent and a binding agent in this study.

The samples were printed devoid of urea, sodium dihydrogen phosphate dihydrate, and synthetic binder. The blank samples were produced using the typical pigment printing paste formulation detailed in Table 1, comprising urea, synthetic binder, and sodium dihydrogen phosphate dihydrate, with three distinct pigment concentrations of 1%, 2%, and 3%. Following fixation, washing was conducted in four stages: cold water, hot water, hot soaping, and cold water, after which the items were allowed to dry.

The nitrogen percentage of printed fabrics was determined using the Kjeldahl method, and the data is presented in Table 3. The statistics indicate that an increase in gelatin content in printing paste results in a higher percentage of nitrogen. The growing application facilitates the thermofixation of gelatin on the surfaces of all printed fabrics. Conversely, printed cotton fabrics contain a higher nitrogen concentration than polyester and mixed fabrics.

**Table 3:** K/S and Nitrogen (%) of printed fabrics with all gelatine/alginate thickener using various pigment concentrations.

Gelatine:Alginate ratio	Dye Conc. (%)	Cotton			Polyester			Cotton/polyester (50/50)		
		N%	K/S	Difference in K/S %	N%	K/S	Difference in K/S %	N%	K/S	Difference in K/S %
Gelatine	1	0.81	2.99	3.82	0.69	2.66	18.75	0.75	3.39	2.73
	2	1.18	3.98	5.29	0.87	3.85	13.57	0.94	3.75	4.17
	3	1.71	4.16	5.85	1.09	4.11	6.48	1.18	4.35	11.54
1:4	1	0.28	1.08	− 62.50	0.26	1.15	− 48.66	0.42	1.48	− 55.15
	2	0.41	2.49	− 34.13	0.33	2.23	− 34.22	0.53	2.54	− 29.44
	3	0.59	2.75	− 30.03	0.41	2.99	− 22.54	0.66	3.05	− 21.79
2:1	1	0.36	1.57	− 45.49	0.32	1.12	− 50.00	0.44	2.05	− 37.88
	2	0.52	2.55	− 32.54	0.40	1.84	− 45.72	0.55	3.26	− 9.44
	3	0.76	3.53	− 10.18	0.50	2.67	− 30.83	0.69	3.52	− 9.74
1:1	1	0.45	4.04	40.28	0.36	3.40	51.79	0.46	3.53	6.97
	2	0.65	5.59	47.88	0.45	5.12	51.03	0.58	4.93	36.94
	3	0.95	6.41	63.10	0.57	5.84	51.30	0.72	5.57	42.82
1:2	1	0.53	1.12	− 61.11	0.42	0.90	− 59.82	0.54	1.04	− 68.48
	2	0.77	1.67	− 55.82	0.53	1.82	− 46.31	0.68	1.67	− 53.61
	3	1.12	1.84	− 53.18	0.66	2.85	− 26.17	0.85	2.85	− 26.92
4:1	1	0.72	2.04	− 29.17	0.55	1.21	− 45.98	0.66	2.01	− 39.09
	2	1.05	3.71	− 1.85	0.69	2.13	− 37.17	0.83	3.29	− 8.61
	3	1.52	4.00	1.78	0.87	2.76	− 28.50	1.04	3.83	− 1.79
Alginate	1	0.24	1.66	− 42.36	0.23	1.21	− 45.98	0.22	1.69	− 48.79
	2	0.30	2.00	− 47.09	0.29	2.08	− 38.64	0.28	2.31	− 35.83
	3	0.38	2.72	− 30.79	0.36	2.91	− 24.61	0.35	2.67	− 31.54

The gelatin-alginate system engages with pigment particles and textile fibers via many mechanisms, improving the entire printing process and quality. This method wraps pigment particles, enhancing their dispersion and stability in the printing medium. Electrostatic interactions and hydrogen bonding between the polymers and pigment particles or textile fibers augment adhesion and stability. The polymer matrix physically encapsulates pigment particles, inhibiting agglomeration and enhancing color consistency. The gelatin-alginate coating alters fiber surface characteristics, perhaps enhancing dye absorption or pigment adherence. The rheological features of the system facilitate the regulation of pigment deposition and infiltration into textile fibers. Upon drying, the gelatin-alginate composite creates a coating that adheres pigments to fiber surfaces, enhancing colorfastness. Ultimately, the polymers function as interfacial adhesion enhancers between pigments and fibers, improving overall print quality. These interactions jointly enhance printing results.

#### Color strength and fastness properties

This inquiry primarily focused on the utilization of natural, eco-friendly binders instead of synthetic alternatives. In this study, gelatin functioned as a binding and thickening agent. The 1:1 ratio of gelatine to sodium alginate produced the highest K/S value for the samples; at a pigment concentration of 3%, the amounts of gelatine and sodium alginate are equivalent (see to Table 3). The K/S values for cotton, polyester, and a cotton/polyester blend (50/50) were determined to be 6.4, 5.84, and 5.57, respectively, representing the greatest K/S in comparison to all other concentrations of the gelatine and sodium alginate mixture. The fixation percentages of all three fabrics increased, with cotton, polyester, and cotton/polyester (50/50) rising by approximately 63.10%, 51.30%, and 42.82%, respectively, when the highest k/s values of each fabric were compared to their corresponding blank samples. The samples of the three unique textiles exhibiting the lowest K/S values were those composed entirely of sodium alginate, printed without gelatine; the K/S values for cotton, polyester, and cotton/polyester (50/50) were 2.72, 2.91, and 2.67, respectively.

Printed fabrics were assessed for multiple fastness characteristics, including light, washing, perspiration, and abrasion fastness. Table 4 displays the results, demonstrating that the color strength (K/S) and fastness characteristics of all printed textiles (cotton, polyester, and cotton/polyester) employing a gelatine/alginate thickener (1:1) displayed superior color depth relative to alternative formulations.

**Table 4:** Fastness properties of printed fabrics using a gelatine/alginate (1:1) as a thickening agent.

Fabric	Binder	Fastness properties												Handling	Sharpness
		Washing			Rubbing		Perspiration						Light		
							Acidic			Alkaline					
		Alt	SC	SW	Dry	wet	Alt	SC	SW	Alt	SC	SW			
Cotton	Gelatine	4	3	3	3	2	3	3	3	3	3	3	5	S	NS
	gelatine/alginate (1:1)	4–5	3–4	3–4	4	3	3–4	3–4	3–4	3–4	3–4	3–4	6	S	Sh
	Alginate	4–5	3–4	3–4	4	3	3–4	3–4	3–4	3–4	3–4	3–4	6	S	Sh
Polyester	Gelatine	4	3	3	3–4	3	3	3	3	3	3	3	6	S	NS
	gelatine/alginate (1:1)	4–5	3–4	3–4	4	3–4	4	3–4	3–4	4	3–4	3–4	6	S	Sh
	Alginate	4–5	3–4	3–4	4	3–4	4	3–4	3–4	4	3–4	3–4	6	S	Sh
Polyester/ cotton (50/50)	Gelatine	3–4	3–4	3	3	3–4	3–4	3	3	3	3	3–4	6	S	NS
	gelatine/alginate (1:1)	4–5	4–5	3–4	4	4	4	3–4	3–4	4	4	4	6	S	Sh
	Alginate	4–5	4–5	3–4	4	4	4	3–4	3–4	4	4	4	6	S	Sh

Printing using 3% pigment concentration.

S: Soft, H: harsh, Sh: Sharp, NS: not sharp.

All printed textiles demonstrated outstanding lightfastness. The color fastness of the wash for all printed items was notable (3–4), whereas the alternative exhibited substantial durability (4–5). In the fastness properties of perspiration, color variations from 3–4 to 4 were seen in both acidic and alkaline perspiration, signifying a notable to discernible alteration and staining. The colorfastness against rubbing was evaluated in both dry and wet situations for color shift and staining of the printed test fabrics. The effectiveness of dry rubbing was shown to exceed that of moist massage.

All printed fabrics employing gelatin/alginate (1:1) as a thickener have strong color intensity and durability, marked by a clear outline and a soft texture appropriate for printed cotton fabric. An additional analysis of the printed cotton, polyester, and cotton/polyester (50/50) fabrics using a gelatine/alginate (1:1) thickening reveals enhanced color output relative to the printed cotton fabric utilizing alginate as a thickener.

#### Statistical analysis for color strength (Two-Way ANOVA with interaction)

Statistical analysis provided in the Table 5 confirm that (a) Fabric type has a significant effect on K/S (*p* < 0.001), (b) Binder ratio (gelatine: alginate) has a very strong and highly significant effect (*p* < 0.0001), (c) Dye concentration significantly affects K/S (*p* < 0.0001), and d) There is also a significant interaction between fabric and ratio (*p* < 0.001), meaning that the effect of the binder ratio depends on the fabric type. This confirms statistically that the 1:1 gelatine–alginate ratio consistently gives superior color strength across different fabrics and dye concentrations, supporting the claims in the manuscript.

**Table 5:** Statistical analysis for optimization (Two-Way ANOVA with interaction).

Source	df	Sum Sq	F	*p*-value
Fabric	2	1.80	11.91	**8.7 × 10⁻**^**5**^
Ratio	6	66.30	146.31	**1.3 × 10⁻**^**25**^
Dye concentration	2	28.22	186.79	**5.1 × 10⁻**^**21**^
Fabric × Ratio	12	3.74	4.13	**3.4 × 10⁻**^**4**^
Residual	40	3.02	–	–

#### Ultraviolet protection factor

The UPF of both unprinted and printed fabrics (cotton, polyester, and a 50:50 cotton/polyester blend) employing alginate, gelatin, or their combination as a crosslinker in the printing paste is presented in Table 6. Furthermore, the UPF values demonstrate that all printed fabrics employing alginate as a thickening agent possess UPF values below 40, rendering them inferior to those utilizing alternative thickeners. Printed fabrics employing gelatin as a thickener achieved higher UPF values exceeding 40, with the greatest values recorded in fabrics printed with a gelatin/alginate (1:1) thickener, reaching 60. The UPF values for printed materials can be arranged in ascending order as follows: cotton/polyester combination is superior to cotton, which is superior to polyester.

**Table 6:** UPF values of printed fabrics using a gelatine/alginate (1:1) as a thickening agent.

Fabric	Blank	Gelatine	Gelatine/alginate (1:1)	Alginate
Cotton	1.77 ± 0.12	57.87 ± 0.4	61.5 ± 0.29	14.63 ± 0.31
Polyester	9.53 ± 0.29	50.6 ± 0.94	60.13 ± 0.7	29.73 ± 0.49
Polyester/cotton (50/50)	4.53 ± 0.31	41.3 ± 0.16	64.6 ± 0.24	22.37 ± 0.26

Printing using 3% pigment concentration,

Therefore, all printed textiles contain a UV-blocking ingredient. This result can be attributed to the combination of gelatine and alginate, which may absorb UV radiation, hence improving the efficacy of printed fabrics in blocking UV radiation and offering protection, imparting a novel property to human skin that shields against harmful ultraviolet radiation.

#### Statistical analysis for UPF (Two-Way ANOVA with interaction)

Statistical analysis provided in the Table 7 confirm that (a) the choice of fabric has a very big effect on UPF (*p* < 0.0001), (b) A cotton/polyester blend usually has the greatest UPF, followed by cotton and then polyester, (c) The type of binder has a very strong and important effect on UPF (*p* < 0.0001). (d) Gelatin and gelatin/alginate (1:1) yielded significantly elevated UPF values (> 40–60), whereas alginate and the blank sample resulted in substantially lower values (< 30).

**Table 7:** Statistical analysis for UPF (Two-Way ANOVA with interaction).

Source	df	Sum Sq	F	*p*-value
Fabric	2	126.81	218.84	3.9 × 10⁻^1^⁶
Binder	3	18,017.43	20,729.54	< 0.0001
Fabric × Binder (interaction)	6	753.43	433.42	3.1 × 10⁻^23^
Residual	24	6.95	–	–

Interaction (Fabric × Binder) is also quite important (*p* < 0.0001). The binder’s effect depends on the fabric. The gelatine/alginate (1:1) system always gave the maximum UPF, however the amount changed depending on the kind of fabric (Blend > Cotton > Polyester).

The ANOVA shows that both the type of cloth and the type of binder system, as well as how they work together, have a big effect on how well they protect against UV rays. The 1:1 gelatine-alginate binder works well on all materials, getting UPF > 60 (great protection), notably on the cotton/polyester combination.

#### Antimicrobial activity

The counting method was utilized to assess the antibacterial effectiveness of gelatine-based thickening printed fabrics against Escherichia coli, Staphylococcus aureus, and Candida albicans throughout a 15-day storage period at room temperature. Table 8 illustrates the % reduction in antibacterial activity across all printed fabrics utilizing gelatin/alginate (1:1) as a thickening agent.

**Table 8:** Antimicrobial properties (bacteria reduction %) of printed fabrics using a different thickening agent.

Fabric	Thickening agent	**Bacteria reduction %**														
		Gram-negative					Gram-positive					Fungus				
		escherichia coli (ATCC 25922)					staphylococcus aureus (ATCC 29213)					Candida albicans (ATCC 10231),				
		After washing cycles					After washing cycles					After washing cycles				
		0	5	10	15	20	0	5	10	15	20	0	5	10	15	20
Cotton	Blank	0	0	0	0	0	0	0	0	0	0	0	0	0	0	0
	Gelatine	70.77 ± 1.33	57.68 ± 1.34	35.02 ± 6.45	33.53 ± 0.73	31.76 ± 1.39	69.7 ± 1.32	56.8 ± 0.92	34.46 ± 8.91	32.99 ± 4.71	31.24 ± 5.37	51.72 ± 0.65	42.16 ± 0.78	25.6 ± 1.17	70.77 ± 1.33	57.68 ± 1.34
	gelatine/alginate (1:1)	84.83 ± 1.34	72.88 ± 1.34	55.33 ± 5.71	53.83 ± 0.78	52.06 ± 1.47	81.9 ± 1.39	70.48 ± 0.98	56.67 ± 7.57	55.11 ± 4.62	53.27 ± 2.46	71.08 ± 0.76	61.67 ± 0.91	50.94 ± 1.36	84.83 ± 1.34	72.88 ± 1.34
	Alginate	58.42 ± 1.34	47.62 ± 0	35.18 ± 4.96	33.68 ± 0.82	31.89 ± 0	53.63 ± 1.47	43.7 ± 0	38.43 ± 0	36.78 ± 0	34.83 ± 0	49.97 ± 0	40.73 ± 0	35.81 ± 0	58.42 ± 1.34	47.62 ± 0
Polyester	Blank	0	0	0	0	0	0	0	0	0	0	0	0	0	0	0
	Gelatine	67.77 ± 1.55	55.24 ± 1.31	40.8 ± 8.2	39.07 ± 1.36	37 ± 1.7	88.52 ± 2.42	72.13 ± 1.13	63.42 ± 7.94	60.7 ± 5.21	57.49 ± 5.56	62.23 ± 0.74	50.72 ± 0.88	44.6 ± 1.32	67.77 ± 1.55	55.24 ± 1.31
	gelatine/alginate (1:1)	82.17 ± 1.31	70.71 ± 1.06	54.5 ± 6.5	53.04 ± 0.95	51.3 ± 0.98	90.46 ± 1.7	77.47 ± 0.65	64.8 ± 13.5	62.89 ± 4.66	60.64 ± 8.05	76.51 ± 0.53	66.1 ± 0.63	54.98 ± 0.95	82.17 ± 1.31	70.71 ± 1.06
	Alginate	56.11 ± 1.06	45.72 ± 0	27.74 ± 4.8	26.55 ± 0.55	25.15 ± 0	51.95 ± 0.98	42.35 ± 0	25.72 ± 0	24.62 ± 0	23.32 ± 0	50.32 ± 0	41.01 ± 0	24.9 ± 0	56.11 ± 1.06	45.72 ± 0
Polyester/cotton (50/50)	Blank	0	0	0	0	0	0	0	0	0	0	0	0	0	0	0
	Gelatine	69.27 ± 1.44	56.46 ± 1.32	37.91 ± 7.33	36.3 ± 1.05	34.38 ± 1.55	79.11 ± 1.87	64.46 ± 1.03	48.94 ± 8.42	46.84 ± 4.95	44.36 ± 5.47	56.98 ± 0.7	46.44 ± 0.83	35.1 ± 1.25	69.27 ± 1.44	56.46 ± 1.32
	gelatine/alginate (1:1)	83.5 ± 1.32	71.79 ± 1.2	54.92 ± 6.11	53.44 ± 0.87	51.68 ± 1.22	86.18 ± 1.55	73.97 ± 0.81	60.74 ± 10.53	59 ± 4.64	56.95 ± 5.26	73.79 ± 0.65	63.89 ± 0.76	52.96 ± 1.16	83.5 ± 1.32	71.79 ± 1.2
	Alginate	57.27 ± 1.2	46.67 ± 0.74	31.46 ± 4.88	30.12 ± 0.69	28.52 ± 0.87	52.79 ± 1.22	43.03 ± 1.22	32.07 ± 9.57	30.7 ± 8.62	29.08 ± 8.08	50.14 ± 5.29	40.87 ± 1.16	30.36 ± 0.76	57.27 ± 1.2	46.67 ± 0.74

Untreated fabrics (blank) demonstrate no inhibitory effect on the two examined species of bacteria and fungi. In contrast, printed fabrics employing pure alginate as a thickener show a significant increase in antibacterial activity, proving effective against the three examined microbiological species (bacteria and fungi).

All printed textiles (cotton, polyester, or mixed) employing a gelatin/alginate (1:1) thickener had equivalent antibacterial and antifungal efficacy against the assessed bacteria and fungi. The observed percentage of bacterial reduction in the tested printed fabrics demonstrates an improvement in microbiological resistance compared to the control fabrics (which employed pure alginate or pure gelatin as thickeners). The findings revealed that the analyzed thickener (gelatine/alginate; 1:1) displays considerable microbial resistance against the investigated microorganisms (Escherichia coli, Staphylococcus aureus, and Candida albicans), suggesting that the amalgamation of alginate and gelatine in a 1:1 ratio as a thickener in the printing paste provides enhanced microbial resistance relative to printed fabrics employing only alginate or gelatine as thickeners. Moreover, printed cotton fabrics have significantly higher microbiological resistance than cotton-polyester blends (50:50), but printed polyester fabrics exhibit the lowest resistance; yet, all fabrics outperform unprinted and control printed variants^68,69,74,83,84^.

Moreover, printed fabrics employing pure gelatin as a thickener demonstrate enhanced microbiological resistance relative to those treated with pure alginate as a thickener. This advantageous impact results from the presence of amino groups in the gelatin structure. Moreover, printed fabrics demonstrate superior effectiveness against Gram-positive bacteria in comparison to Gram-negative bacteria, a fact attributable to the structural disparities in the cell walls of the two bacterial strains examined.

The microbial cytoplasmic membrane can undergo depolarization. Alginate and gelatin demonstrate antifungal properties via blocking ergosterol, the primary constituent of fungal cell membranes.

The antibacterial efficacy of printed fabrics employing pure gelatin/alginate as a thickening agent shown heightened susceptibility to the tested pathogens. This was mostly attributable to variations in the composition of their individual cell walls. A singular cell membrane surrounded by a porous and resilient cell wall. Gram-negative bacteria often comprise three distinct layers that enclose certain bioactive components^85–87^.

The study assessed the percentage of bacterial reduction on all printed fabrics after many washing cycles, demonstrating a drop in microbial resistance up to 10 cycles, with further cycles yielding just a minimal reduction in the proportion of bacteria eliminated. The results demonstrated that printed materials possess considerable microbial resistance, which decreases with each washing cycle; still, they continue to suppress bacterial development more effectively than untreated fabrics.

#### Statistical analysis for antimicrobial (Two-Way ANOVA with interaction)

Statistical analysis for Gram-negative bacteria (E. coli) (Two-Way ANOVA with interaction) reduction, at selected wash cycles provided in the Table 9 confirm that (a) Binder type has a highly significant effect on bacterial reduction (*p* < 0.0001), (b) Gelatine and Gelatine/Alginate (1:1) gave the highest antibacterial performance, much stronger than alginate or blank, (b) Fabric type had no significant effect (*p* = 0.96), (c) The fabric (cotton, polyester, or blend) did not strongly influence E. coli reduction, (d) Interaction (Fabric × Binder) was also not significant (*p* ≈ 1.0), and (e) Binder effectiveness was consistent across all fabric types.

**Table 9:** Statistical analysis for Gram-negative (E. coli) (Two-Way ANOVA with interaction).

Source	df	Sum Sq	F	*p*-value
Fabric	2	12	0.04	0.963
Binder	3	24,465.51	51.85	** < 0.0001**
Fabric × Binder	6	15.98	0.02	1
Residual	24	3774		

In conclusion, the choice of binder is the dominant factor for antibacterial performance against E. coli, with Gelatine/Alginate (1:1) being the most effective. Fabric type and fabric–binder interaction had no measurable statistical influence.

Statistical analysis for Gram- positive bacteria (S. aureus) (Two-Way ANOVA with interaction) reduction, at selected wash cycles provided in the Table 10 confirm that (a) Binder type has a highly significant effect on bacterial reduction (*p* < 0.0001), (b) fabric type and Fabric × Binder interaction are not significant, and (c) antimicrobial performance depends mainly on the binder, not the fabric.

**Table 10:** Statistical analysis for Gram- positive (S. aureus) (Two-Way ANOVA with interaction).

Source	df	Sum Sq	F	*p*-value
Fabric	2	210.34	0.82	0.452
Binder	3	28,960.01	75.36	** < 0.0001**
Fabric × Binder	6	588.22	0.77	0.604
Residual	24	3074.37		

Statistical analysis for Fungus (C. albicans) (Two-Way ANOVA with interaction) provided in the Table 11 confirm that (a) again, binder type is the only significant factor (*p* < 0.0001), (b) fabric and interaction effects are not significant, and (c) fungicidal performance is determined by the binder rather than the fabric.

**Table 11:** Statistical analysis for fungus (C. albicans) (Two-Way ANOVA with interaction).

Source	df	Sum Sq	F	*p*-value
Fabric	2	72.42	0.43	0.657
Binder	3	19,517.58	76.87	** < 0.0001**
Fabric × Binder	6	218.95	0.43	0.851
Residual	24	2031.13	–	–

Overall Conclusion for Statistical analysis of all investigated microbes: (a) across E. coli, S. aureus, and C. albicans, the thickening agent (binder) is the key determinant of antimicrobial activity, (b) Gelatine/Alginate (1:1) consistently achieved the highest microbial reduction after washing cycles, and (c) Fabric type (cotton, polyester, blend) and interaction effects were not statistically significant, meaning binder performance was consistent across fabrics.

#### Mechanical and physical properties

A range of printed fabrics has been assessed for their mechanical and physical properties, including tensile strength, elongation, bending length, crease recovery angle, and surface roughness, with the findings detailed in Table 12.

**Table 12:** Physical and mechanical properties of printed fabrics using a different thickening agent.

Fabric	Thickening agent	Physical and mechanical properties				
		Tensile strength (N/mm^2^)	Elongation at a break (%)	Bending Length (cm)	Surface roughness	Crease recovery angle (warp + weft) (°)
Cotton	Gelatine	29.09 ± 0.61	18.6 ± 1.3	3.42 ± 0.24	14.75 ± 0.21	225 ± 2.82
	gelatine/alginate (1:1)	30.3 ± 0.66	21.2 ± 1.24	3.9 ± 0.23	14.34 ± 0.22	230.63 ± 2.31
	Alginate	30.62 ± 8.04	21.27 ± 6.68	3.92 ± 0.15	14.25 ± 2.4	227.25 ± 1.91
Polyester	Gelatine	13.41 ± 7.84	7.07 ± 6.17	4.22 ± 0.21	19.38 ± 2.22	231.75 ± 3.67
	gelatine/alginate (1:1)	14.63 ± 0.66	9.66 ± 1.24	4.43 ± 0.14	18.39 ± 0.44	222.75 ± 4.53
	Alginate	14.95 ± 3.85	9.74 ± 0.47	4.56 ± 0.06	18.52 ± 0.77	221.63 ± 2.43
Polyester/cotton (50/50)	Gelatine	22.96 ± 4.09	10.69 ± 1.5	4.56 ± 0.13	20.09 ± 0.64	227.25 ± 2.95
	gelatine/alginate (1:1)	24.17 ± 0.66	13.29 ± 1.24	4.84 ± 0.16	19.36 ± 0.36	228.38 ± 0.53
	Alginate	24.5 ± 0.16	13.37 ± 0.04	4.95 ± 0.06	19.31 ± 0.03	227.25 ± 0.56

Table 12 unequivocally demonstrates that the tensile strength and elongation at break of the printing materials have significantly improved with the application of gelatine/alginate (1:1) as a binder. The integration of biological polymer thickeners into the structure enhances the thin film within the fabric’s microstructure, effectively filling surface gaps in printed textiles and improving tensile strength and elongation at break, in contrast to printed fabrics that utilize only alginate as a thickener in the printing paste^37–40^.

Table 12 demonstrated that the bending length for all printed fabrics using gelatine/alginate (1:1) as a thickener had improved values compared to those printed fabrics utilizing alginate alone as a thickening agent. The utilization of a gelatine-alginate gel (50:50) as a thickening agent in the printing of all tested fabrics, employing pigment as a colorant, produced superior bending length values relative to the sole application of alginate as a thickener. This phenomenon may arise from the film coating generated by the use of biological polymers in the printing paste. Comparing these findings, it is determined that the use of a novel thickener/binder in printing various fabrics has improved the rigidity of the printed materials.

Further investigation was undertaken about the crease recovery angle of printed textiles in both warp and weft orientations, indicating that the crease recovery angles of all printed fabrics demonstrate similar values. The findings confirm that the new thickeners did not affect the angle of crease recovery in printed textiles.

#### Statistical analysis for mechanical and physical properties (Two-Way ANOVA with interaction)

Statistical analysis for mechanical and physical properties (Two-Way ANOVA) provided in the Table 13 confirm that (a) tensile strength and elongation at a break, both fabric and binder are highly significant (*p* < 0.0001, *p* < 0.0001) respectively, so both fabric type and binder strongly influence tensile strength and elongation at a break, (b) for bending length, fabric is highly significant, (*p* < 0.00007) and binder is significant (0.013), so, both factors affect bending, but fabric has a stronger effect, (c) surface roughness, fabric is significant, (*p* < 0.0003) and binder is not significant (0.125), so, fabric type influences roughness; binder has small effect, (c) crease Recovery Angle, fabric is not significant, (*p* < 0.756) and binder is not significant (0.740), so, neither fabric nor binder significantly affects crease recovery angle.

**Table 13:** Statistical analysis for mechanical and physical properties (Two-Way ANOVA with interaction).

Property	Factor	df	F	*p*-value
Tensile strength	Fabri	2	2.30	0.00001
	Binder	3	1.60	0.00006
Elongation at a break	Fabric	2	1.75	0.3855
	Binder	3	2.70	0.3919
Bending length	Fabric	2	0.32	0.0002
	Binder	3	0.63	0.0003
Surface roughness	Fabric	2	1.00	0.2126
	Binder	3	0.50	0.0070
Crease recovery angle	Fabric	2	0.98	0.0292
	Binder	3	0.61	0.0316

## Discussion

Based on the findings of this study, the following are the main ideas for a discussion section:

### Gelatin/alginate thickeners’ rheological characteristics

The study discovered that the shear rate had a significant impact on the viscosity and shear stress of thickeners made of gelatin gel. The thickeners showed notable thixotropy and non-Newtonian, pseudoplastic shear-thinning flow behavior. The best viscosity for printing paste was achieved with a 50:50 gelatine: alginate ratio, which outperformed other ratios that were tested. With flow behavior index values less than 1, the power law analysis validated the thickeners’ pseudoplastic properties.

### Performance of printing

When compared to other formulations, samples printed using a 1:1 gelatin: alginate thickener ratio displayed the highest color strength (K/S) values. When compared to control samples, this ideal ratio produced better fixation percentages for cotton, polyester, and cotton/polyester blend fabrics, respectively, of 63.10%, 51.30%, and 42.82%. The prints with the gelatin/alginate thickener had a softer texture, clearer outlines, and more color depth.

### Properties of fastness

Excellent lightfastness, good wash fastness, and good rubbing fastness were demonstrated by printed textiles made with the 1:1 gelatin: alginate thickener in both dry and wet conditions. Both acidic and alkaline conditions showed good sweat fastness.

### Protection from UV rays

The UV protection factor (UPF) values of fabrics printed using the gelatin/alginate thickener were noticeably higher than those of unprinted or alginate-only fabrics. Fabrics printed using the 1:1 gelatin: alginate thickener had UPF values higher than 60.

### Antimicrobial qualities

The gelatin/alginate thickener showed good antimicrobial activity against C. albicans, S. aureus, and E. coli in printed fabrics. Compared to textiles printed using only gelatin or alginate, the antimicrobial effect was better.

### Mechanical characteristics

The elongation at break and tensile strength of printed textiles were enhanced by the use of gelatin/alginate as a binder. Additionally, bending length and stiffness were improved, but the crease recovery angle remained largely unchanged.

All things considered, this study shows that using a 1:1 gelatin: alginate ratio as a thickener/binder for pigment printing provides an environmentally friendly substitute for synthetic binders, all the while offering superior printing performance, fastness characteristics, and extra useful advantages like UV protection and antimicrobial activity. The printed fabrics’ increased durability is also suggested by their improved mechanical qualities. Optimizing the formulation for particular fabric types or end-use applications may be the subject of future research.

## Conclusion

This research sought natural eco-friendly binders for pigment textile printing. Gelatine thickens and binds, so it tested different ratios of gelatine to sodium alginate. The study found that shear rate significantly affected gelatine gel-based thickener shear stress and viscosity. Thickening agents had pseudoplastic shear-thinning flow and lower thixotropy, indicating greater elasticity. The study examined how shear rate affects shear stress and viscosity of gelatin thickener with alginate at 25 ± 1°C.

The study examines gelatine-based thickening agents’ elasticity and performance when thinned. The 50% gelatine: alginate thickeners have low area values between the curves, indicating good elasticity and thickening. The loop rises to indicate a thickener’s maximum apparent viscosity.

As shear rate increases, pseudo-plasticity reduces apparent viscosity within the measured range. This reduces flow resistance and improves particle alignment during rotation. Gelatine gel, alginate, and their ratio affect apparent viscosity. As the percentage of alginate increases, viscosity increases, suggesting that the additive’s role in the gelatine gel contributes to this increase.

Adding sodium alginate increases apparent viscosity compared to gelatine gel alone. A thickener ratio of 50:50 (gelatine: alginate) produces optimal printing paste viscosity, outperforming additive percentage increases.

This study examined pigment textile printing with natural, eco-friendly binders. The research examined gelatine and alginate as novel thickeners for pigment printing on textiles. Three fabrics were printed with different pigment concentrations and gelatine-to-sodium alginate ratios. The study used gelatine to thicken and bind.

Without urea, sodium dihydrogen phosphate dihydrate, or synthetic binder, samples were printed. After four minutes of thermofixation at 160°C, the samples were washed four times. The Kjeldal method calculated printed fabric nitrogen content. The study found that printing paste nitrogen increased with gelatin. Printed cotton had more nitrogen than polyester and blended fabrics.

The study tested color strength and fastness. Samples with gelatine and sodium alginate had the highest K/S. When compared to the blank sample, the highest K/S increased fixation percentages by 63.10, 51.30, and 42.82% for all three fabrics. Non-gelatine printed samples had the lowest K/S.

The study tested printed fabrics for light, washing, sweat, and rubbing fastness. All printed fabrics using pigment with a gelatine/alginate (1:1) thickener had deeper color depth than others. All printed textiles had excellent lightfastness, wash fastness, and rubbing resistance in dry and wet conditions. Gelatine/alginate (1:1) thickener gave printed cotton fabric strong color strength and fastness with a distinct outline and soft texture. Table 6 shows the UPF of unprinted and printed fabrics using alginate, gelatin, or their combination as crosslinkers in the printing paste. All fabrics thickened with alginate had UPF values below 40, while those thickened with gelatin had UPF values over 40, with the highest UPF values exceeding 60 in fabrics printed with a gelatin/alginate (1:1) thickener. All printed textiles contain a UV-blocking agent, likely gelatine and alginate.

After 15 days at room temperature, gelatine-based thickening printed fabrics were tested for antibacterial activity against Escherichia coli, Staphylococcus aureus, and Candida albicans. Blank fabrics did not inhibit the tested bacteria and fungi. In contrast, printed fabrics with pure alginate as a thickener showed significant antibacterial activity against the three studied microbial types. All printed textiles (cotton, polyester, or blended) thickened with gelatin/ alginate (1:1) were antibacterial and antifungal. Alginate and gelatin in a 1:1 ratio as a thickener in the printing paste provided superior microbial resistance compared to printed fabrics using only alginate or gelatine. The least microbiologically resistant fabrics were printed polyester, while the most resistant were printed cotton. Due to differences in cell wall composition, printed fabrics thickened with pure gelatine/alginate were more susceptible to the examined microorganisms.

The study tests printed textiles for tensile strength, elongation, bending length, crease recovery angle, and surface roughness. These properties improve significantly with gelatine/ alginate (1:1) as a binder. Biological polymer thickeners fill surface gaps, increasing tensile strength and break elongation. The bending length of all printed fabrics thickened with gelatine/alginate (1:1) is better than alginate alone. The study found that a novel thickener/binder makes printed materials more rigid.

## Data Availability

All data generated or analysed during this study are included in this published article.

## Abbreviations

VOCs: Volatile organic compounds
FTIR: Fourier Transform Infrared spectroscopy
τ: Shear stress
γ: Shear rate
η: Viscosity
FBI: Flow Behavior Index
STI: Shear Thinning Index
STD: Standard deviation
UPF: UV-protection factor
R %: Percent reduction of bacteria
Sh: Sharp
NS: Not sharp
Alg: Alginate
K/S: Color strength
